# A Proteomic Approach for Comprehensively Screening Substrates of Protein Kinases Such as Rho-Kinase

**DOI:** 10.1371/journal.pone.0008704

**Published:** 2010-01-14

**Authors:** Mutsuki Amano, Yuta Tsumura, Kentaro Taki, Hidenori Harada, Kazutaka Mori, Tomoki Nishioka, Katsuhiro Kato, Takeshi Suzuki, Yosuke Nishioka, Akihiro Iwamatsu, Kozo Kaibuchi

**Affiliations:** 1 Department of Cell Pharmacology, Graduate School of Medicine, Nagoya University, Nagoya, Japan; 2 Department of Cardiology, Graduate School of Medicine, Nagoya University, Nagoya, Japan; 3 Protein Research Network, Inc., Yokohama, Japan; 4 Japan Science and Technology Agency, CREST, Kawaguchi, Japan; INMI, Italy

## Abstract

**Background:**

Protein kinases are major components of signal transduction pathways in multiple cellular processes. Kinases directly interact with and phosphorylate downstream substrates, thus modulating their functions. Despite the importance of identifying substrates in order to more fully understand the signaling network of respective kinases, efficient methods to search for substrates remain poorly explored.

**Methodology/Principal Findings:**

We combined mass spectrometry and affinity column chromatography of the catalytic domain of protein kinases to screen potential substrates. Using the active catalytic fragment of Rho-kinase/ROCK/ROK as the model bait, we obtained about 300 interacting proteins from the rat brain cytosol fraction, which included the proteins previously reported as Rho-kinase substrates. Several novel interacting proteins, including doublecortin, were phosphorylated by Rho-kinase both *in vitro* and *in vivo*.

**Conclusions/Significance:**

This method would enable identification of novel specific substrates for kinases such as Rho-kinase with high sensitivity.

## Introduction

Protein phosphorylation is one of the most ubiquitous and essential mechanisms mediating intracellular signal transduction in various cellular processes. About 500 protein kinases are encoded in the human genome, where these are mainly divided into two groups, Ser/Thr protein kinases and Tyr protein kinases. Kinases recognize and phosphorylate their specific substrates and modulate their functions. In the cell, numerous proteins are continuously and dynamically phosphorylated and dephosphorylated under the control of complex signaling networks. Comprehensive screening of substrates for kinases is necessary to increase understanding of the signaling networks in which protein kinases participate. However, it remains difficult to efficiently screen the physiological substrates of protein kinases.

*In vitro* kinase assays have been used to identify potential substrates for specific kinases for many years. As an extension of this method, genome-wide screening of substrates for 87 yeast protein kinases has been performed using protein microarrays containing 4,400 yeast proteins [1]. However, this method requires a large number of recombinant proteins, and the native conformation of substrates may be lost on the plates. One of the recent phosphoproteomic strategies is the semi-quantitative liquid chromatography tandem mass spectrometry (LC-MS/MS) approach combined with phosphopeptide enrichment, in which proteins or peptides from cells treated with agonists and protein kinase inhibitors are labeled with stable isotope or isobaric reagent iTRAQ ([2], [3] for reviews). Two-dimensional fluorescence difference gel electrophoresis (2D-DIGE) has also been used to identify potential substrates for ERK from the cells treated with a MEK inhibitor [4]. Both methods require specific antagonists, agonists and/or RNA interference to identify the responsible kinases. Thus, screening of direct substrates for specific kinases is still laborious and difficult.

Protein kinases share common catalytic domain structures composed of a small N-terminal lobe and a large C-terminal lobe. The cleft between these lobes is the active center that binds to both ATP and the substrate. In spite of highly analogous structures, protein kinases exhibit striking substrate specificity partly due to their surface charge and hydrophobicity [5]. In addition to the active center, several kinases, such as MAPK, GSK3 and PDK1, have been reported to associate with substrates through extra docking sites, which may confer substrate specificity and facilitate phosphorylation efficiency [6]. Nevertheless, the interaction between protein kinases and substrates is transient and not very stable, such that utilizing the interaction to identify substrates has been thought to be difficult, with a few exceptions. However, recent improvement in the sensitivity of mass spectrometry is expected to make it possible to detect substrate proteins weakly associated with the catalytic domain of protein kinases.

Here, we developed a method combining affinity column chromatography, using the active catalytic fragment of protein kinase as a bait, and shotgun LC-MS/MS to efficiently screen the kinase substrates. We employed Rho-kinase/ROCK/ROK, a Ser/Thr protein kinase belonging to the AGC family of kinases, as a model protein kinase. Rho-kinase is an effector of small GTPase Rho and is implicated in various cellular functions, including cell migration, cell adhesion, smooth muscle contraction, cytokinesis and neurite retraction [7], [8]. Here, we describe our discovery of more than a hundred proteins that specifically interacted with Rho-kinase, some of which functioned as Rho-kinase substrates.

## Results

### Affinity column chromatography of Rho-kinase

**Figure 1:**
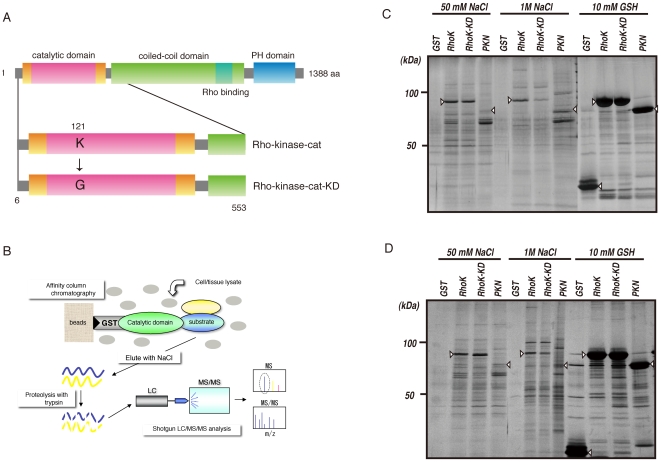
Isolation of interacting proteins for the catalytic domain of Rho-kinase. (A) Domain structure of Rho-kinase and the constructs used for affinity column chromatography. (B) Strategy for isolation of protein kinase substrates. (C, D) Isolation of Rho-kinase-cat-interacting proteins from rat brain cytosol (C) and P2 (D) fractions. The cytosolic or P2 fraction of rat brain lysate was loaded onto a Glutathione-Sepharose column coated with either GST, GST-Rho-kinase-cat, GST-Rho-kinase-cat-KD or GST-PKN-cat. The bound proteins were eluted by addition of 1 M NaCl after washing with 50 mM NaCl. The eluates were analyze by SDS-PAGE, and visualized by silver staining. Arrowheads indicate the GST-tagged proteins used as baits.

To screen potential substrates of Rho-kinase, we examined whether the active catalytic fragment of Rho-kinase (Rho-kinase-cat) interacts with its substrates by affinity column chromatography. Rat brain cytosol or peripheral membrane (P2) fractions concentrated by ammonium sulfate precipitation were loaded onto a glutathione-sepharose affinity column on which GST, GST-Rho-kinase-cat, or GST-Rho-kinase-cat-KD, a kinase-deficient mutant of Rho-kinase, was immobilized (Figure 1A, B). GST-PKN-cat, another Rho effector belonging to the PKC subfamily in the AGC family of kinases, was also subjected to affinity column chromatography. The proteins bound to the affinity columns were then eluted by addition of 50 mM and 1 M NaCl, and then 10 mM glutathione. Numerous proteins were detected in the eluates from the GST-Rho-kinase-cat, GST-Rho-kinase-cat-KD and GST-PKN-cat columns (Figure 1C, D). The apparent pattern of eluted proteins in the eluate from the GST-Rho-kinase-cat column was similar to that from the GST-Rho-kinase-cat-KD column, and different from that off the GST-PKN-cat column.

### Identification and phosphorylation of Rho-kinase-interacting proteins

**Figure 2:**
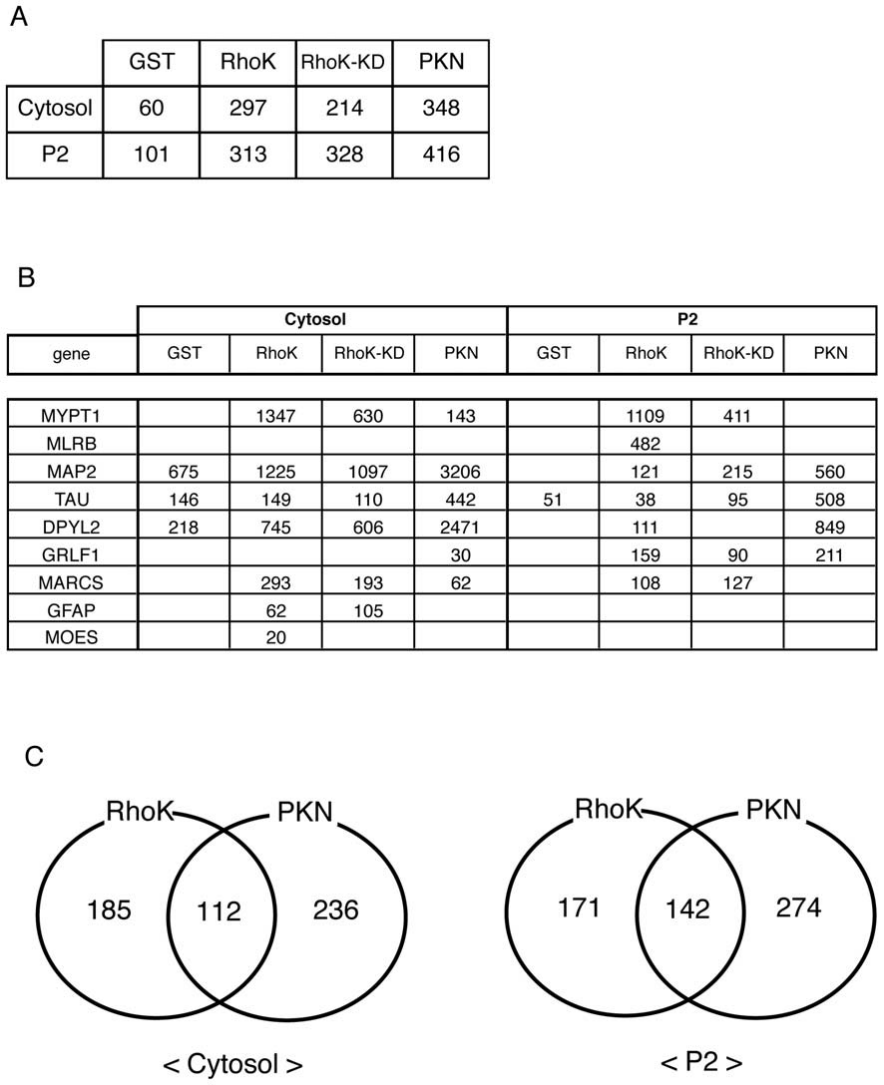
Identification of Rho-kinase-cat-interacting proteins. (A) Total numbers of hit proteins detected in eluates off each affinity column. (B) Known substrates for Rho-kinase detected in eluates off each affinity column. The numbers indicate protein scores by Mascot analysis. (C) The numbers of proteins specifically or commonly detected in GST-Rho-kinase-cat and GST-PKN-cat columns are shown. These results are representatives of at least three independent experiments.

The proteins eluted off the affinity columns with 1 M NaCl were subjected to reduction, alkylation, demineralization, concentration, and then digestion with trypsin. The peptide mixtures were subjected to reverse phase HPLC and subsequent mass spectrometry. Measurements of peptide masses by LC-MS/MS were repeated several times under different conditions. Lists identified representative Rho-kinase-cat-interacting proteins from the rat brain cytosol and P2 fractions are shown in Supplementary Table, which lists 297 and 313 identified proteins. Under the same conditions, 60, 214, and 348 proteins from the cytosol fraction, and 101, 328, and 416 proteins from the P2 fraction were identified in the eluates from the GST, GST-Rho-kinase-cat-KD, and GST-PKN-cat affinity columns, respectively (Figure 2A). Several known substrates, such as MYPT1 and CRMP-2 (DPYL2), were detected in the eluate from the GST-Rho-kinase-cat column (Figure 2B). MYPT1 was specifically detected from the GST-Rho-kinase-cat and -cat-KD columns, but was barely observable from the GST-PKN-cat column, whereas CRMP-2 was also detected in eluate off the GST-PKN-cat column with higher scores (Figure 2B). The numbers of proteins specifically or commonly detected off of GST-Rho-kinase-cat and -PKN-cat columns are shown in Figure 2C. To confirm whether the previously identified substrates exist in these samples, immunoblot analysis was performed. The bands recognized by anti-CRMP-2 Ab were detected in the 1M NaCl eluates from the GST-Rho-kinase-cat, -cat-KD and -PKN-cat columns and were in good agreement with their protein scores from LC-MS/MS analysis (Figure S1). Mascot scores are calculated for every peptide and protein, and indicate the absolute probability that observed match is a random event, demonstrating that high score is a low possibility. The more number peptides are identified, the higher score of the protein. Although it is not a quantitative evaluation, protein scores are expected to partly reflect the abundance of proteins, which was consistent with our immunoblot data.

**Figure 3:**
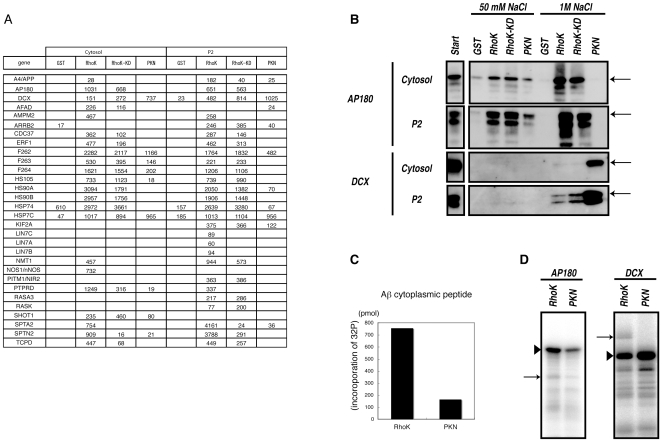
Identification of novel substrates for Rho-kinase. (A) Protein scores of APP, AP180, and DCX in eluates off each affinity column. (B) Immunoblot analysis of eluates with anti-AP180 and -DCX Abs. Eluates off affinity columns with 50 mM and 1 M NaCl were subjected to immunoblot analysis with anti-AP180 and -DCX Abs. AP180 was detected in both 50 mM and 1 M NaCl eluates off the Rho-kinase-cat column, but was barely detectable in eluates off the PKN-cat column. On the contrary, DCX was strongly detected in eluates off the PKN-cat column, and moderately off the Rho-kinase-cat column. Arrows indicate the positions of AP180 and DCX. The lower bands are supposed to be degradation products or splice variants. (C) The APP cytoplasmic peptide was incubated with GST-Rho-kinase-cat or GST-PKN-cat in the presence of 100 µM [γ-^32^P] ATP for 1 h at 30°C. The reaction mixtures were applied to P81 paper and subjected to scintillation counting. (D) GST-AP180 (left) or GST-DCX (right) was incubated with GST-Rho-kinase-cat or GST-PKN-cat in the presence of 100 µM [γ-^32^P] ATP for 1 h at 30°C. The reaction mixtures were subjected to SDS-PAGE, and phosphorylated proteins were imaged by autoradiography. Arrowheads and arrows indicate substrates and autophosphorylation of Rho-kinase, respectively. These results are representatives of at least three independent experiments.

In addition to the known substrates, a number of proteins specifically or preferentially associated with Rho-kinase (Figure 3A). Of note, Rho-kinase-cat-interacting proteins included transmembrane proteins, such as amyloid precursor protein (APP) and the receptor-type tyrosine-protein phosphatase delta (PTPRD) (Figure 3A). This may be explained by the brain extract including the microsomal fraction. Among novel Rho-kinase-cat-interacting proteins, APP, AP180, and Doublecortin (DCX) were subjected to further analyses as candidates of novel substrates because they represent transmembrane, endocytic and cytoskeletal proteins. APP and AP180 were detected in eluates from the Rho-kinase-cat column, but were barely detectable in eluates from the PKN-cat column, whereas DCX was detected in eluates from the PKN-cat column, with higher scores than the Rho-kinase-cat columns (Figure 3A). The elution profiles of AP180 and DCX were also examined by immunoblot analysis, which demonstrated that the profiles were similar to LC-MS/MS scores (Figure 3B). To investigate whether these proteins are phosphorylated by Rho-kinase, an in vitro phosphorylation assay was performed. The cytoplasmic region of APP and GST-AP180 were efficiently phosphorylated by Rho-kinase-cat, and weakly by PKN-cat (Figure 3C, D). GST-DCX was efficiently phosphorylated by both Rho-kinase-cat and PKN-cat. Phosphorylation levels of GST-DCX by PKN-cat were slightly higher than those by Rho-kinase-cat (Figure 3D). We also confirmed that CRMP-2 was phosphorylated by PKN-cat (data not shown). Phosphorylation efficacy of these proteins appeared to correlate with their affinities for the respective catalytic fragments of Rho-kinase and PKN. These results suggest that this method, based on kinase-substrate interaction, is effective for identifying new substrates.

### Phosphorylation of DCX by Rho-kinase

**Figure 4:**
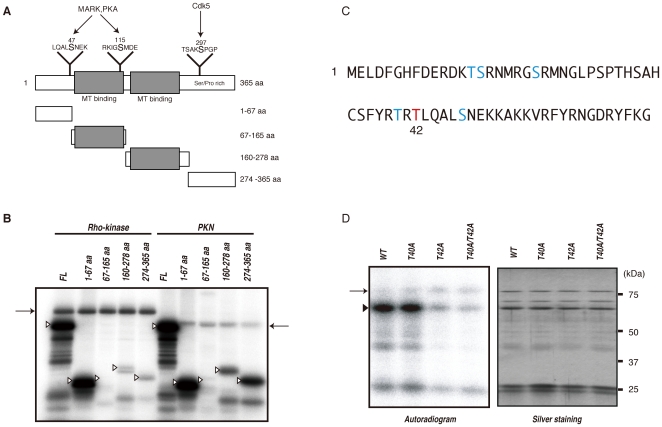
Identification of phosphorylation sites of DCX by Rho-kinase. (A) Schematic representation of the domain structures and deletion mutants of DCX. Sites phosphorylated by MARK, PKA and Cdk5 are also shown. (B) Phosphorylation of DCX deletion mutants. The indicated GST-DCX fragments were phosphorylated by GST-Rho-kinase-cat or GST-PKN-cat. The phosphorylated proteins were imaged by autoradiography. Open arrowheads and arrows indicate the positions of substrates and autophosphorylation of kinases, respectively. These results are representatives of at least three independent experiments. (C) Sequence of and potential phosphorylation sites within the 1–67 aa region. The major phosphorylation site was identified as Thr42 (*red*). (D) Phosphorylation of DCX mutants with amino acid substitutions. GST-DCX-WT, -T40A, -T42A or -T40A/T42A was incubated with Rho-kinase-cat and 50 µM [γ-^32^P] ATP for 10 min at 30°C. The reaction mixtures were subjected to SDS-PAGE and GST-fused proteins were visualized by silver staining (*right*). Phosphorylated proteins were imaged by autoradiography (*left*). Arrowhead and arrow indicate the positions of substrates and autophosphorylation of kinase, respectively. These results are representatives of at least three independent experiments.

To examine whether this method is useful for screening novel substrates, not only in vitro but also in vivo, we focused on DCX, because the role of DCX and its phosphorylation by PKA and Cdk5 has been elucidated [9], [10]. DCX binds and stabilizes microtubules in young neurons, and Rho regulates the reorganization of actin and microtubules [11]. We narrowed down the phosphorylation sites in DCX by making four fragments of DCX, GST-DCX-N1 (1–67 aa), -N2 (67–165 aa), -C1 (160–278 aa) and -C2 (274–365 aa) (Figure 4A). Among these fragments, Rho-kinase most heavily phosphorylated DCX-N1, indicating that the N1 region includes the major phosphorylation site. PKN also phosphorylated DCX-N1 efficiently, and -C1 and -C2 moderately (Figure 4B). R/KXXS/T or R/KXS/T (X is any amino acid) are known as the consensus phosphorylation sites of Rho-kinase. The potential phosphorylation sites in DCX-N1 are Thr-14, Ser-15, Ser-21, Thr-40 and Thr-42 (Figure 4C). To determine the possible Rho-kinase phosphorylation sites in DCX, we produced GST-DCX-S14A, -S15A, -T21A, -T40A and -T42A, in which serine or threonine was substituted with alanine, and examined phosphorylation efficiency. The phosphorylation level of DCX-T42A by Rho-kinase was much lower than that of GST-DCX-wild type (WT) (Figure 4D). The phosphorylation level of DCX-T40A was slightly lower (Figure 4D), and other mutations of GST-DCX did not affect phosphorylation (date not shown), indicating that Thr-42 is the major phosphorylation site. The phosphorylation level of GST-DCX-T40A/T42A by Rho-kinase was less than that of GST-DCX-T42A (Figure 4D), suggesting that Thr-40 is a minor phosphorylation site. DCX is phosphorylated at Ser-47 by PKA and at Ser-297 by Cdk5 [9], [10]. We also confirmed that Rho-kinase did not phosphorylate DCX at Ser-47 or at Ser-297 (data not shown).

**Figure 5:**
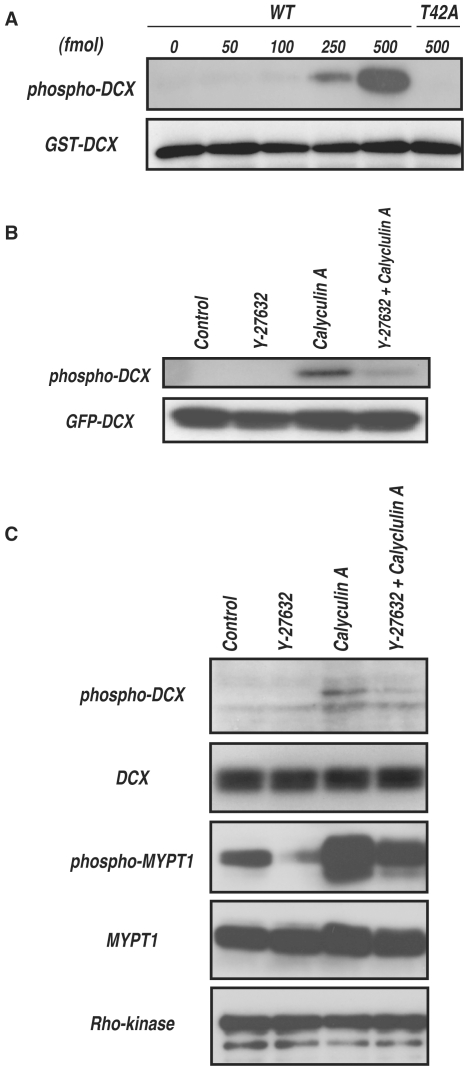
Phosphorylation of DCX *in vivo*. (A) Specificity of the antibody against DCX phosphorylated at Thr-42 (anti-pT42 Ab). One pmol of GST-DCX containing the indicated amounts of phosphorylated GST-DCX-WT or -T42A was subjected to SDS-PAGE, followed by immunoblot analysis with anti-pT42 Ab (upper panel) or anti-GST Ab (lower panel). (B) Phosphorylation of DCX in COS7 cells. GFP-DCX was transiently expressed into COS7 cells. The transfected cells were treated with DMSO or 20 µM Y-27632 for 15 min, and then treated with or without 0.1 µM calyculin A for 10 min. The cell lysates were analyzed by immunoblot analysis with anti-pT42 Ab (upper panel) or anti-GFP Ab (lower panel). These results are representatives of at least three independent experiments. (C) Phosphorylation of DCX in hippocampal neurons at DIV1. The cells were treated with DMSO or 20 µM Y-27632 for 20 min, and then treated with or without 50 nM calyculin A for 7 min. The cell lysates were analyzed by immunoblot analysis with anti-pT42 Ab or anti-DCX Ab. Phosphorylation of MYPT1 was also examined with anti-MYPT1 pT853 Ab. These results are representatives of at least three independent experiments.

To monitor DCX phosphorylation by Rho-kinase, the polyclonal antibody that specifically recognizes DCX phosphorylated at Thr-42 (anti-pT42 Ab) was produced using a phosphopeptide corresponding to amino acids 37–47 of DCX, in which Thr-42 is phosphorylated as an antigen. The immunoblot analysis revealed that anti-pT42 Ab recognized the phosphorylated DCX in a dose-dependent manner, but did not react with the non-phosphorylated form or T42A mutant (Figure 5A).

To examine whether Rho-kinase phosphorylates DCX in intact cells, GFP-DCX was transfected into COS7 cells, because DCX is specifically expressed in young neurons but not in most non-neuronal cell lines, including COS7 cells. The immunoblot analysis showed that phosphorylation of GFP-DCX at Thr-42 is undetectable under the conditions in which GFP-DCX was well expressed (Figure 5B). To induce DCX phosphorylation in COS7 cells, the cells were treated with calyculin A (CLA), a PP1/PP2A-type phosphatase inhibitor [12]. CLA enhanced phosphorylation of GFP-DCX, and Y-27632, a specific inhibitor of Rho-kinase, inhibited CLA-induced phosphorylation, suggesting that Rho-kinase can phosphorylate GFP-DCX at Thr-42 in COS7 cells under certain conditions. GFP-DCX might be constantly phosphorylated and dephosphorylated in COS7 cells. To explore DCX phosphorylation under more physiological conditions, we employed rat hippocampal young neurons that expressed DCX. DCX is thought to play a critical role in neuronal migration by regulating microtubules downstream of Reelin, which also acts upstream of Rho [11]. CLA enhanced phosphorylation of endogenous DCX, and Y-27632 inhibited CLA-induced phosphorylation (Figure 5C), suggesting that Rho-kinase can phosphorylate DCX in neurons. Nevertheless, we cannot exclude the possibility of the off-target effects of Y-27632 on other kinases, including PKN.

### Effect of phosphorylation of DCX at the Rho-kinase site on microtubule organization in HeLa cells

**Figure 6:**
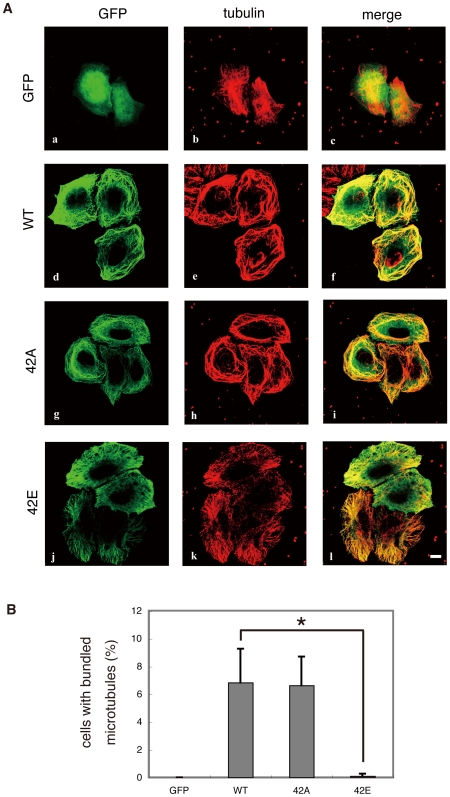
Effect of mutations of DCX at phosphorylation sites on microtubule organization in HeLa cells. (A) Effect of overexpression of DCX-WT, -T42A and -T42E in HeLa cells. DCX mutants with substitutions at phosphorylation sites were expressed in HeLa cells. HeLa cells expressing GFP-DCX or its mutants were fixed in methanol for 10 min at room temperature. After washing, the cells were immunostained with anti-GFP and anti-tubulin Abs. Colors indicate GFP (green) and tubulin (red). These results are representatives of at least three independent experiments. Scale bar, 10 µm. (B) The percentages of cells with highly bundled microtubules. Data are means ± SD of three independent experiments. Asterisks indicate that there is a significant difference from the value of control cells (p<0.05).

Phosphorylation of DCX by Cdk5 or PKA decreases the ability of DCX to bind to microtubules [9], [10]. To examine the effects of phosphorylation of DCX at the Rho-kinase site, DCX mutants with a substitution at the phosphorylation site were expressed in HeLa cells (Figure S2). GFP was diffusely distributed in HeLa cells (Figure 6A, a). GFP-tagged DCX-WT and the -T42A mutant were localized to microtubules (Figure 6A, d and g) and caused highly bundled microtubules in a certain population of cells (Figure 6A, e and h, and 6B), whereas GFP-DCX-T42E, a phospho-mimicking form, was still localized to the microtubules (Figure 6A, j), but lost its ability to bundle microtubules (Figure 6A, k, and 6B), suggesting that phosphorylation of DCX at Thr-42 weakens its microtubule-bundling activity.

## Discussion

In this study, we found that combining affinity column chromatography using the catalytic domain of Rho-kinase and shotgun LC-MS/MS analysis is powerful for identification of substrates, including known substrates and novel ones, such as DCX, APP and AP180. We also found that Rho-kinase phosphorylates DCX not only in vitro, but also in vivo. Thus, compared to previous methods, our method can easily enrich substrates for specific kinases and can be applied for screening other weak enzyme-substrate interactions. One of the advantages of this method is the ability to screen protein kinase substrates, including transmembrane proteins, in their native states. However, it lacks information concerning direct phosphorylation, phosphorylation sites, or in vivo phosphorylation. Combining this simple method with a conventional method, such as 2D-DIGE, would be more informative and powerful for screening novel substrates of specific kinases and would increase understanding of the phosphorylation network.

We employed Rho-kinase and PKN as model kinases, both of which belong to the Rho effector and AGC families with similar kinase domains. Nevertheless, Rho-kinase-cat-interacting proteins were quite different from those of PKN-cat (Figure 1C, D, Supplementary Table S1), suggesting that the proteins obtained by affinity column chromatography bind specifically to the respective catalytic domains. Among the known substrates, MYPT1 was detected in eluates off the Rho-kinase-cat affinity column with much higher scores than off the PKN-cat affinity column. AP180 and APP were enriched in the Rho-kinase-cat affinity column. MAP2, Tau and CRMP-2, as well as DCX, were detected in both columns, but the scores in the Rho-kinase-cat affinity column were lower than those in the PKN-cat affinity column. Tau is phosphorylated by PKN [13] as well as by Rho-kinase [14]. We confirmed that CRMP-2 and DCX were efficiently phosphorylated by PKN in vitro. It can be speculated that the binding affinities to the catalytic domain might reflect the phosphorylation efficiency. Of note, when we used GSK3β, which belongs to the CMGC kinase family with a kinase domain distinct from that of the AGC family, as a bait, the apparent pattern of proteins off the GSK3β affinity column on a SDS-PAGE gel is completely different than that using Rho-kinase (data not shown).

We also found that more CRMP-2 was found in the 50 mM NaCl eluate from the Rho-kinase-cat column than in the 1 M NaCl eluate. AP180 was also detected in 50 mM NaCl eluates, whereas DCX was not. Because kinase-substrate interaction is predicted to be weak and the dissociation is faster, analysis of eluates with low ionic strength would be also informative.

Rho-kinase-cat-KD-interacting proteins largely overlapped with Rho-kinase-cat-interacting proteins, including both known and newly identified substrates, suggesting that catalytic activity of protein kinases is not essential for kinase-substrate interaction. Nonetheless, it is noteworthy that several proteins, such as nNOS and brain spectrin, specifically associated with Rho-kinase-cat, but not with Rho-kinase-cat-KD; thus, the interaction of Rho-kinase-cat with a certain group of proteins may be activity-dependent. The other possibility is that the latter group associates with Rho-kinase-cat only through the active center, whereas the group interacting with both Rho-kinase-cat and -cat-KD associates with Rho-kinase-cat at multiple sites.

The necessity of ATP for the binding to substrates is dependent on kinases. Kinetic analyses of protein kinase revealed that there are two kinetic mechanisms to form a ternary complex of kinase, substrate, and ATP: a random sequential binding mechanism and an ordered sequential binding mechanism. Rho-kinase has been shown to bind randomly to ATP and substrates, whereas certain types of kinase such as protein kinase A have been reported to bind ATP first [15], [16]. Actually, we confirmed that apparent bands on SDS PAGE in eluate off Rho-kinase-cat affinity column and interaction of AP180 with Rho-kinase-cat were not increased in the presence of ATP (data not shown). However, ATP would be necessary to add to the column for kinases which show an ordered sequential binding with ATP first. Further analysis is underway to optimize the conditions and to understand the relationship between binding properties and phosphorylation efficiency.

## Materials and Methods

### Materials and chemicals

A rabbit polyclonal antibody against DCX phosphorylated at Thr-42 (anti-pT42 Ab) was produced against the phosphopeptide Cys-Phe^37^-Tyr-Arg-Thr-Arg-phosphoThr^42^-Leu-Gln-Ala-Leu-Ser^47^ by Biologica Co. (Osaka, Japan). GST, GST-Rho-kinase-cat (6–553 amino acids), GST-Rho-kinase-cat-KD (Lys121, which is essential for ATP-binding, is replaced by Gly), and GST-PKN-cat (581–942 amino acids) were produced in Sf9 cells with a baculovirus system and purified on a glutathione-sepharose column [17]. The cDNAs encoding DCX were kindly provided by Dr. Nakagawa (University of California). Trypsin for mass spectrometry was purchased from Promega Co (Madison, WI). Cy2- or Cy3-conjugated secondary antibodies against mouse or rabbit immunoglobulin G were purchased from Jackson ImmunoResearch Laboratories (West Grove, PA, USA). Other materials and chemicals were obtained from commercial sources.

### Plasmid construction

Human DCX (full length), DCX-N1 (1–67 aa), DCX-N2 (67–165 aa), DCX-C1 (160–278 aa) and DCX-N2 (274–365 aa) were amplified by the polymerase chain reaction (PCR) and subcloned into the pGEX-2T (GE Healthcare, Princeton, NJ, USA) and/or pEGFP-C1 (Clontech Laboratories, Mountain View, CA, USA) plasmids. The cDNAs of human DCX-T14A, DCX-S15A, DCX-S21A, DCX-T40A, DCX-T42A, DCX-S47A and DCX-T40A/T42A, in which Ala is substituted for Thr-14, Ser-15, Ser-21, Thr-40, Thr-42, Ser-47 and Thr-40/Thr-42, were generated by site-directed mutagenesis and subcloned into the pGEX-2T and/or pEGFP-C1 plasmids. GST-fusion proteins were produced in *E.coli* and purified on a glutathione-sepharose column.

### Affinity column chromatography

The affinity chromatography was performed essentially as previously described [18], [19]. The P5–7 rat brain cytosol fraction or the P2 fraction prepared by ammonium sulfate precipitation (0–80%) was loaded onto a 250 µl-glutathione-sepharose affinity column, on which 5 nmol of GST, GST-Rho-kinase-cat, GST-Rho-kinase-cat-KD or GST-PKN-cat was immobilized. After washing the columns three times with 850 µl of buffer A (20 mM Tris/HCl, pH 7.5, 1 mM EDTA, 1 mM DTT) and three times with 850 µl of buffer A containing 50 mM NaCl, the bound proteins were subsequently eluted three times with 850 µl of buffer A containing 1 M NaCl and three times with 850 µl of buffer A containing 10 mM glutathione. The first eluate was evaluated by SDS-PAGE, and proteins were detected by silver staining.

### Mass spectrometry

The proteins in the eluate were digested by trypsin for 16 h at 37°C after reduction, alkylation, demineralization, and concentration. Nanoelectrospray tandem mass analysis was performed using a Finnigan LTQ/Orbitrap mass spectrometry (ThermoFisher Scientific Inc., Waltham, MA) system combined with a MAGIC2002 HPLC System (Michrom BioResources Inc., Auburn, CA). Samples were injected onto the MAGIC2002 HPLC System equipped with a MonoCap column 0.1 mm in diameter and 50 mm in length (AMR Inc., Tokyo Japan). Reversed-phase chromatography was performed with a linear gradient (0 min, 5% B; 45min, 100% B) of solvent A (2% acetonitrile with 0.1% trifluoroacetic acid) and solvent B (98% acetonitrile with 0.1% trifluoroacetic acid) at an estimated flow rate of 500 nl/min. The mass spectrometer was equipped with a XYZ interface (AMR Inc., Tokyo, Japan). Ionization was performed by a PicoTip, 20 µm in diameter (New Objective Inc., Woburn, MA) with a capillary voltage at 2.5 kV and temperature of 200°C. A precursor ion scan was carried out using a 250–4500 mass to charge ratio (m/z) prior to MS/MS analysis. Multiple MS/MS spectra were submitted to the program Mascot (Matrix Science Inc., Boston, MA) for the MS/MS ion search.

### Phosphorylation

The phosphorylation assay was performed as previously described [18]. In brief, the kinase reactions of Rho-kinase on the APP cytoplasmic peptide (40 µM), GST-AP180 (1 µM), GST-DCX (1 µM) and GST-DCX fragments (1 µM) were carried out in 50 µl of a reaction mixture (50 mM Tris/HCl, pH 7.5, 1 mM EDTA, 1 mM EGTA, 1 mM DTT, 5 mM MgCl_2_, 100 µM [γ-^32^P] ATP [1 to 20 GBq/mmol]), 0.1 µM purified GST-Rho-kinase-cat or GST-PKN-cat, and substrates for 1 h at 30°C. Then, the reaction mixtures were spotted onto p81 paper for APP cytoplasmic peptide, or boiled in SDS sample buffer and subjected to SDS-PAGE for GST-fusion proteins.

The kinase reactions of Rho-kinase on the GST-DCX A mutants were carried out in 100 µl of a reaction mixture (50 mM Tris/HCl, pH 7.5, 1 mM EDTA, 1 mM EGTA, 1 mM DTT, 5 mM MgCl_2_, 50 µM [γ-^32^P] ATP [1 to 20 GBq/mmol]), 0.1 µM purified GST-Rho-kinase-cat, and purified GST-DCX A mutants (0.1 µM) for 10 min at 30°C. Then, the reaction mixtures were boiled in SDS sample buffer and subjected to SDS-PAGE. The radiolabeled peptide or proteins were analyzed by scintillation counting or by an image analyzer (BAS1500; Fuji, Tokyo, Japan).

### Cell culture and immunoblot analysis

COS7 cells were seeded on a 6-well dish at a density of 1.5×10^5^ cells/well in Dulbecco's modified Eagle medium (DMEM) with 10% fetal bovine serum (FBS) at 37°C in an air/5% CO_2_ atmosphere at constant humidity. Transfections were carried out using the Lipofectamine reagent (Invitrogen) according to the manufacturer's protocol. Cells were grown in DMEM with 10% FBS for one day. Rat hippocampal neurons were prepared as previously described [20]. In brief, 8×10^5^ neurons were seeded on a 35-mm plastic dish coated with poly-D-lysine and laminin, and cultured for one day. Cells were treated with dimethyl sulfoxide (DMSO) or Y-27632, and then treated with or without calyculin A. The cells were collected with SDS sample buffer and subjected to SDS-PAGE and immunoblot analysis using an anti-GFP Ab, an anti-DCX Ab or an anti-pT42 Ab.

### Cell culture and immunostaining

HeLa Cells were seeded on a 13-mm glass coverslip coated with poly-D-lysine at a density of 5×10^3^ in DMEM with 10% FBS and cultured overnight at 37°C in an air/5% CO_2_ atmosphere at constant humidity. Transfections were carried out using the Lipofectamine reagent (Invitrogen), according to the manufacturer's protocol. Cells were grown in DMEM with 10% FBS for one day. Cells were fixed with methanol for 10 min at room temperature. Cells were washed with PBS at room temperature. Cells were then incubated with anti-GFP and anti-α-tubulin antibodies for 60 min at room temperature. After washing, the samples were incubated with a Cy3- or Cy2-conjugated secondary antibody. Fluorescence was examined using a Zeiss LSM 5 PASCAL laser confocal microscope (Carl Zeiss, Obeokochem, Germany).

## Supporting Information

Figure S1 Detection of CRMP-2 (DPYL2) in Rho-kinase-cat-interacting proteins. Eluates off affinity columns with 50 mM and 1 M NaCl were subjected to immunoblot analysis with anti-CRMP-2 Ab. CRMP-2 was strongly detected in eluates off the PKN-cat column, and moderately off the Rho-kinase-cat column. Middle panel is a longer exposure of the same blot shown in upper panel.
(0.50 MB TIF)

Figure S2 Expression of GFP-DCX in HeLa cells. HeLa cell lysate expressing GFP, GFP-DCX-WT, or GFP-DCX mutants was subjected to immunoblot analysis with anti-GFP Ab. The expression levels of GFP-DCX mutants were almost same as that of GFP-DCX-WT.
(0.25 MB TIF)

Table S1 Representative list of kinase-interacting proteins detected by affinity column chromatography and LC-MS/MS analysis.
(0.24 MB XLS)
