# Supplementary figures and images for: A Proteomic Approach for Comprehensively Screening Substrates of Protein Kinases Such as Rho-Kinase
Source: PLoS One. 2010 Jan 14;5(1):e8704. doi: 10.1371/journal.pone.0008704 (PMC2806833; doi:10.1371/journal.pone.0008704)

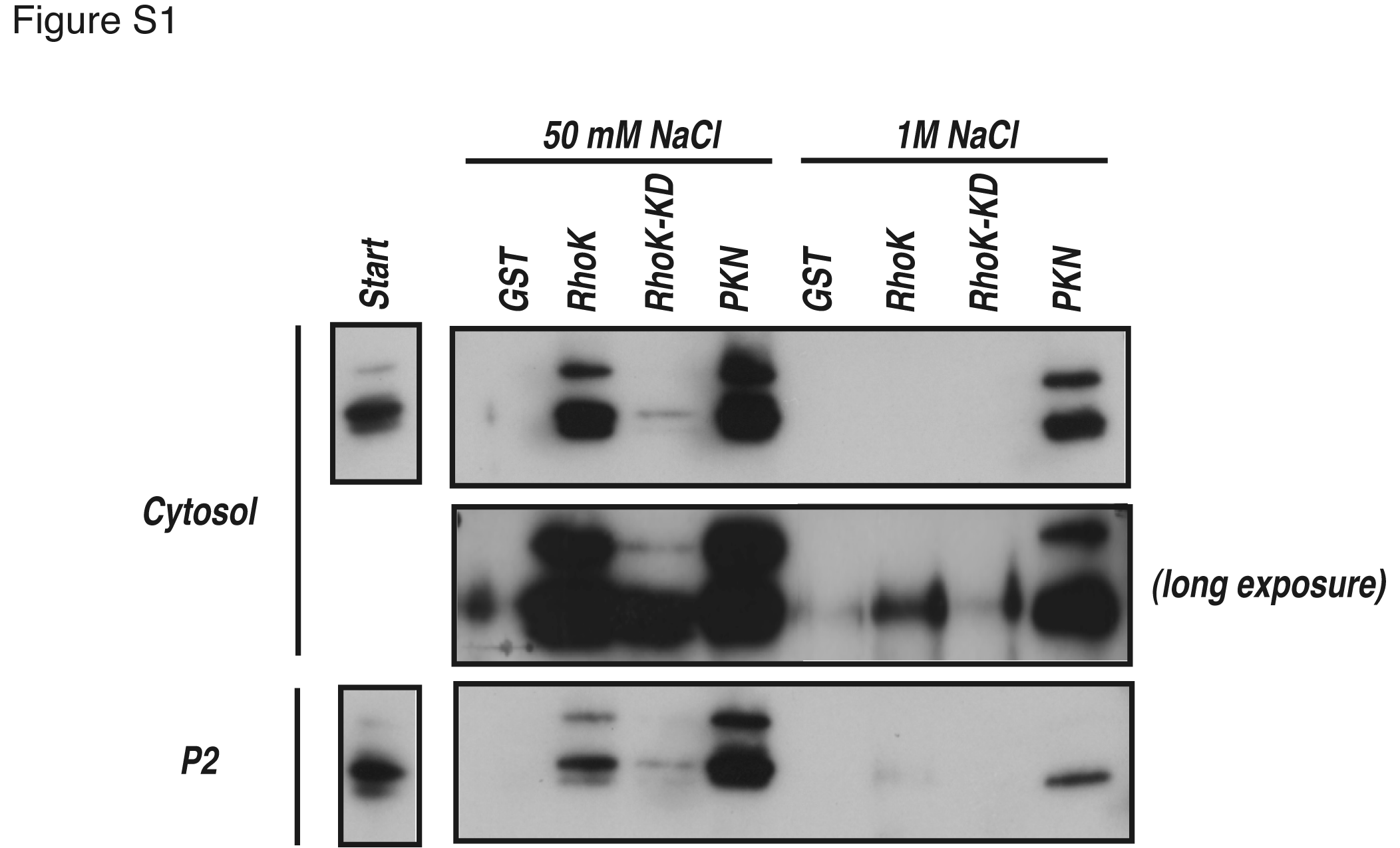

Supplement: Figure S1 — Detection of CRMP-2 (DPYL2) in Rho-kinase-cat-interacting proteins. Eluates off affinity columns with 50 mM and 1 M NaCl were subjected to immunoblot analysis with anti-CRMP-2 Ab. CRMP-2 was strongly detected in eluates off the PKN-cat column, and moderately off the Rho-kinase-cat column. Middle panel is a longer exposure of the same blot shown in upper panel. (0.50 MB TIF) [file pone.0008704.s001.tif]

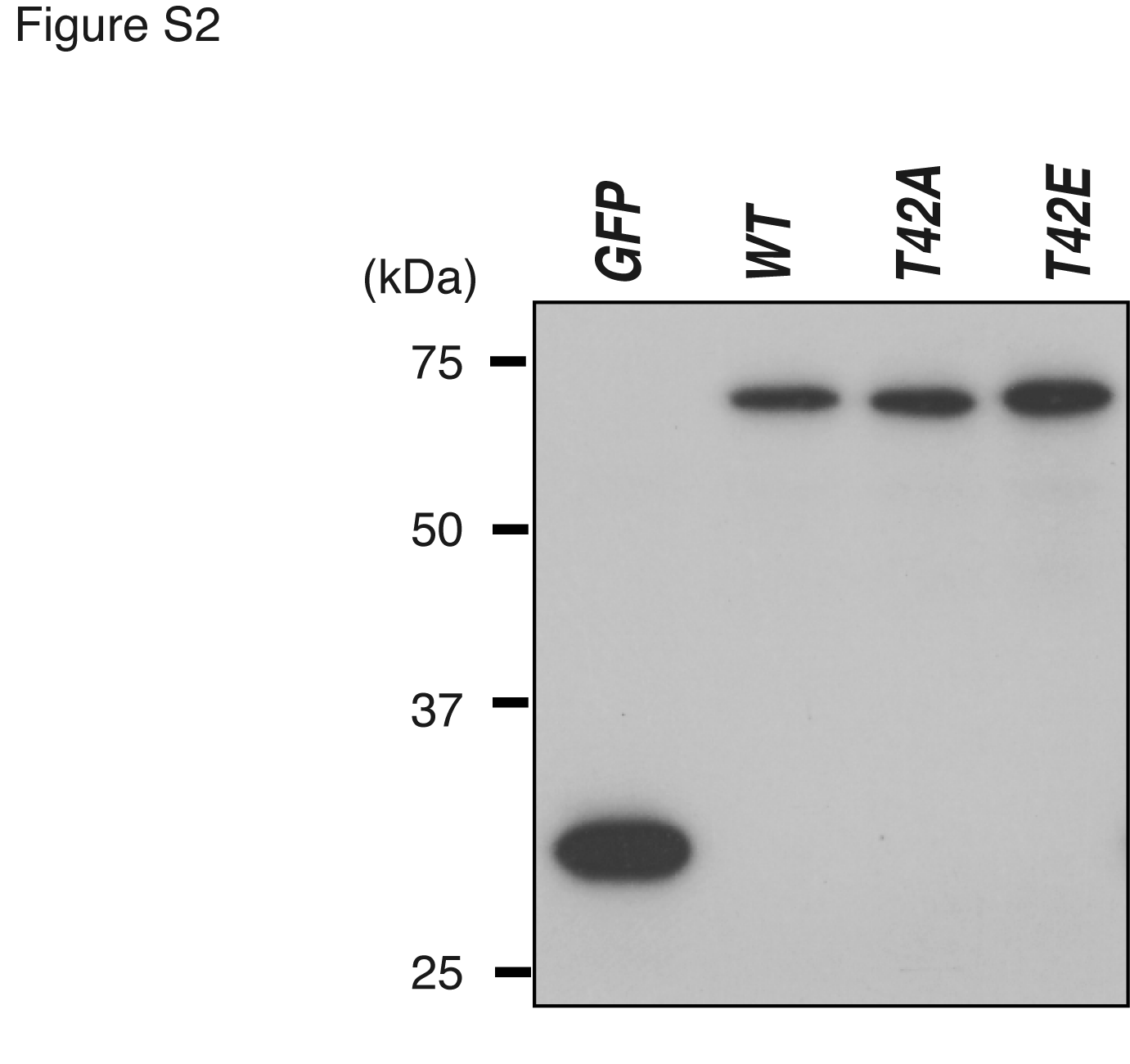

Supplement: Figure S2 — Expression of GFP-DCX in HeLa cells. HeLa cell lysate expressing GFP, GFP-DCX-WT, or GFP-DCX mutants was subjected to immunoblot analysis with anti-GFP Ab. The expression levels of GFP-DCX mutants were almost same as that of GFP-DCX-WT. (0.25 MB TIF) [file pone.0008704.s002.tif]
